# Supplementary material for: Extracellular Vesicle-Based Bronchoalveolar Lavage Fluid Liquid Biopsy for EGFR Mutation Testing in Advanced Non-Squamous NSCLC
Source: Cancers (Basel). 2022 May 31;14(11):2744. doi: 10.3390/cancers14112744 (PMC9179452; doi:10.3390/cancers14112744)
Supplement: Supplementary file 1 [file cancers-14-02744-s001.zip › cancers-1710292-supplementary.pdf]

Supplementary Table S1. Comparison of EGFR genotyping in matched tissue, BALF and plasma liquid biopsy (n=110).

		Tissue (N=110)	
		EGFR mutant type	Wild type
		66	44
BALF (N=110)	EGFR mutant type	65	0
	Wild type	1	44
Plasma (N=110)	EGFR mutant type	32	6
	Wild type	34	38
